# Transcranial Direct Current Stimulation Modulates Human Color Discrimination in a Pathway-Specific Manner

**DOI:** 10.3389/fpsyt.2012.00078

**Published:** 2012-09-12

**Authors:** Thiago L. Costa, Balázs V. Nagy, Mirella T. S. Barboni, Paulo S. Boggio, Dora F. Ventura

**Affiliations:** ^1^Laboratório da Visão, Experimental Psychology Department, University of São PauloSão Paulo, Brazil; ^2^Social and Cognitive Neuroscience Laboratory and Developmental Disorders Program, Center for Health and Biological Sciences, Mackenzie Presbyterian UniversitySão Paulo, Brazil

**Keywords:** color vision, koniocellular pathway, parvocellular pathway, V1, tDCS, transcranial direct current stimulation

## Abstract

Previous research showed that transcranial direct current stimulation (tDCS) can modulate visual cortex excitability. However, there is no experiment on the effects of tDCS on color perception to date. The present study aimed to investigate the effects of tDCS on color discrimination tasks. Fifteen healthy subjects (mean age of 25.6 ± 4.4 years) were tested with Cambridge Color Test 2.0 (Trivector and ellipses protocols) and a Forced-choice Spatial Color Contrast Sensitivity task (vertical red-green sinusoidal grating) while receiving tDCS. Anodal, cathodal, and sham tDCS were delivered at Oz for 22 min using two square electrodes (25 cm^2^ with a current of 1.5 mA) in sessions separated by 7 days. Anodal tDCS significantly increased tritan sensitivity (*p* < 0.01) and had no significant effect on protan, deutan, or red-green grating discrimination. The effects on the tritan discrimination returned to baseline after 15 min (*p* < 0.01). Cathodal tDCS reduced the sensitivity in the deutan axis and increased sensitivity in the tritan axis (*p* < 0.05). The lack of anodal tDCS effects in the protan, deutan, and red-green grating sensitivities could be explained by a “ceiling effect” since adults in this age range tend to have optimal color discrimination performance for these hues. The differential effects of cathodal tDCS on tritan and deutan sensitivities and the absence of the proposed ceiling effects for the tritan axes might be explained by Parvocellular (P) and Koniocellular (K) systems with regard to their functional, physiological, and anatomical differences. The results also support the existence of a systematic segregation of P and K color-coding cells in V1. Future research and possible clinical implications are discussed.

## Introduction

Color vision is a popular model system for information processing in neural circuits and human color perception has been successfully used as a model to assess the functional status of the central nervous system (Gobba and Cavalleri, 2003; Ventura et al., 2003, 2004, 2005, 2007; Silva et al., 2005; Costa et al., 2006, 2007; Feitosa-Santana et al., 2008, 2010; Moura et al., 2008; Barboni et al., 2009; Conway et al., 2010). Current understanding of the human color perception system can be considered extensive when compared to our understanding of other sensory systems. On the other hand several relevant unanswered questions remain, especially concerning the organization and tuning of color-coding cells in V1 and the organization of color processing pathways in the extrastriate visual cortex. The variety of congenital and acquired color vision defects and the lack of effective rehabilitative procedures are also noteworthy. As pointed by Simunovic (2010), the current management of congenital color vision deficiency is mostly limited to career counseling although animal experiments point to a future for gene therapy (Mancuso et al., 2009).

To date, the possibility of modulating human color vision using transcranial non-invasive neuromodulatory techniques was not yet evaluated. Techniques such as transcranial direct current stimulation (tDCS) can complement current research by introducing a causal approach in which the effects of inhibitory and excitatory interventions over a specific brain area can be evaluated in a specific task. Several lines of research in neuroscience benefited from using this rationale (for reviews see Nitsche et al., 2008; Zaghi et al., 2010).

Transcranial direct current stimulation is a non-invasive brain modulation technique that uses weak direct currents with polarity-dependent functional effects: cathodal currents being generally inhibitory while anodal being excitatory (Nitsche and Paulus, 2000; Nitsche et al., 2008). In the past 10 years researchers were successful in using tDCS to modulate human visual system performance (Antal and Paulus, 2008). Significant results include improvements in luminance contrast sensitivity (Antal et al., 2001, 2004a; Accornero et al., 2007), phosphene threshold reduction (Antal et al., 2003a,b), sensitivity in central visual field measured by standard automated perimetry (Kraft et al., 2010), and different visuomotor skills (Antal et al., 2004b,c; Bolognini et al., 2010a,b). In addition, tDCS has modulatory effects on multisensory integration tasks (Bolognini et al., 2010a, 2011) and illusory phenomena (Varga et al., 2007; Bolognini et al., 2011).

The use of tDCS as a tool for stroke patient’s rehabilitation is promising since these patients show improvements in visual system performance even after one single tDCS session (Ko et al., 2008; Halko et al., 2011). Similarly in congenital and acquired color vision deficiencies tDCS might be used to improve the remaining color discrimination performance. Furthermore, gene therapy is quickly advancing as a potential treatment for congenital color deficiency, but if applied in humans it will probably be accompanied by behavioral training (Mancuso et al., 2009). In this panorama tDCS could be a valuable tool to boost the behavioral outcomes of the treatments.

If tDCS can affect color perception, future research applying tDCS to the visual cortex during visual discrimination tasks should take into account the color parameters of the stimuli used. When taken together, the abovementioned arguments justify the urgent and crucial nature of the current investigation. In the present study we examined the effect of tDCS on color discrimination thresholds and chromatic contrast thresholds using current psychophysical methodology. Considering the literature on tDCS modulation of visual perception, we hypothesize that tDCS will have a significant effect on color discrimination.

## Materials and Methods

### Participants and study design

We conducted a randomized, single-blind repeated-measures study to evaluate the effects of tDCS delivered to the visual cortex on color discrimination thresholds and on chromatic contrast thresholds. Fifteen healthy subjects (mean age of 25.6 ± 4.4 years) with no history of neuropsychiatric or visual system disorders participated in this study. Subjects had no metallic implants and were not under treatment with medication that could affect central nervous system function and were not smokers or users of psychoactive drugs. All participants had normal or corrected to normal visual acuity (Snellen 20/20).

**Figure 1:**
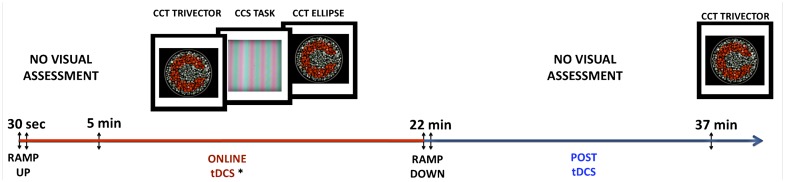
**Summary of experimental procedures**. tDCS current was ramped up during the first 30 s of the procedure. Participants received 5 min of tDCS before starting the visual assessment. In each session, visual tests were performed in a random order. After 22 min of stimulation, the current would be ramped down and the participant would have a 15-min break without performing visual tests. After the break, the tDCS Trivector test was repeated without tDCS.

Participants were submitted to three sessions of tDCS: one for sham stimulation, one for anodal, and one for cathodal stimulation of the visual cortex. The sessions were separated by an interval of 7 days, and all procedures were the same in the three sessions, except for the tDCS modality. The order of the sessions and the order of the visual tests applied in each session were randomized across subjects and across sessions. The sessions for each participant occurred at a similar time of the day to try to avoid eventual confounding factors. The participants received 5 min of tDCS only, followed by 17 min of tDCS during the visual tests, totalizing 22 min of stimulation. Fifteen minutes after the end of the stimulation the participants were tested again with the Cambridge Color Test (CCT) Trivector protocol (see Color Vision Assessment). A summary of the session *procedures* is presented in Figure 1.

The study was approved by the institutional ethics committees of the University of São Paulo Biomedical Sciences Institute (1025/CEP) and Mackenzie Presbyterian University, Brazil, and registered at the National Ethics Committee (SISNEP, Brazil – CAAE – 0097.0.272.134-11). Written informed consent was obtained from all participants.

### Transcranial direct current stimulation

Transcranial direct current stimulation was delivered through two square (25 cm^2^) saline-soaked sponge electrodes connected to a specially developed, battery-driven direct current stimulator with a maximum output of 2 mA. Stimulation intensity was set at 1.5 mA, generating a current density of 0.06 mA/cm^2^. Electrodes were placed at Oz and Cz (according to the International 10-20 EEG System, Jasper, 1958). For anodal stimulation, anode electrode was placed over Oz and the cathode over Cz, while the contrary was true for the cathodal tDCS condition. Non-conductive elastic bandages were used to hold the electrodes in place.

In each session the current was ramped from 0 to 1.5 mA in 10 s. In the sham stimulation condition the current was ramped down after 30 s of stimulation, the equipment’s sham mode was activated and the session was conducted in the same way as the active stimulation sessions. In sham mode the equipment continues working without passing current through the electrodes and all stimulation parameters are visible in the display, resembling an active stimulation condition. By receiving 30 s of stimulation the participant can feel the initial skin sensation associated with active tDCS but the stimulation is considered ineffectual for neuromodulation purposes. This procedure is considered efficient for blinding subjects with respect to stimulation parameters (Gandiga et al., 2006).

### Color vision assessment

Color vision was assessed with two computer based psychophysical tests: Cambridge Color Test 2.0 (Cambridge Research Systems) and a Forced-choice Spatial Chromatic Contrast Sensitivity task (CCS) developed by our group. Both tests ran on a Dell microcomputer and the stimuli were presented through a VSG 2/5 Visual Stimulus Generator in a Viewsonic G90fB 19″ CRT monitor. The monitor’s gamma correction was done immediately before the beginning of the research using an Optical 200E Photometer (Cambridge Research Systems). Both tests were performed binocularly in a dark room with the participants seated 3 m away from the monitor screen and using a remote control (CT6 model, Cambridge Research Systems).

**Figure 2:**
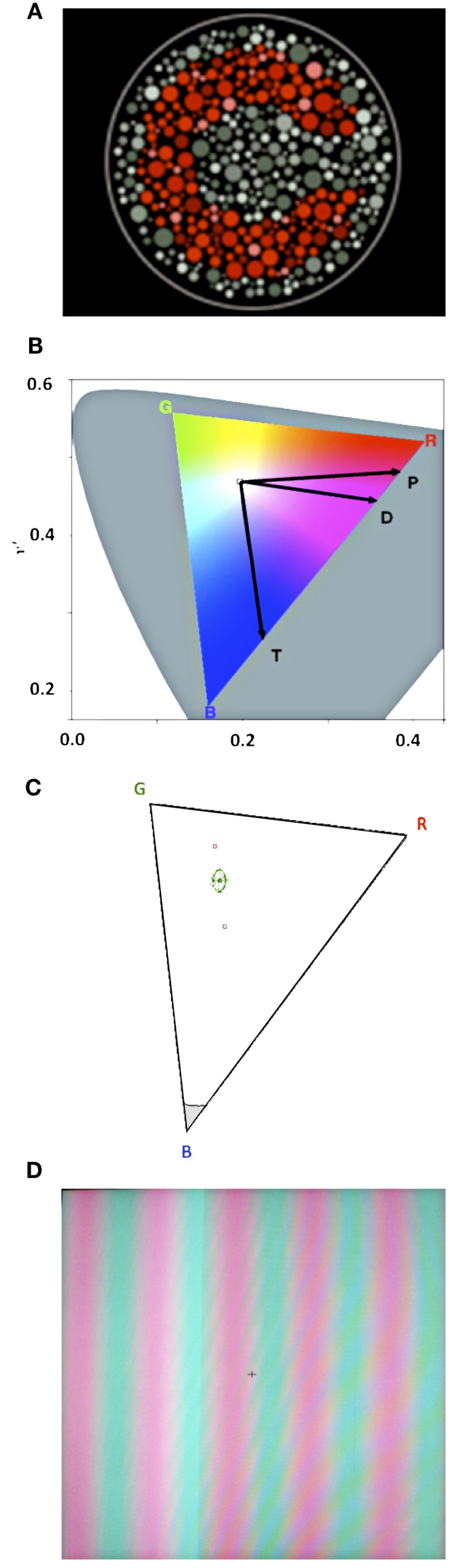
**(A)** Example of the pseudoisochromatic adaptation of Landolt’s C used in the Cambridge Color Test 2.0. **(B)** CIE 1976 color space with color confusion axes. “P” stands for protan, “D” stands for deutan, and “T” stands for tritan. The color triangle represents the monitor’s color gamut within the CIE 1976 color space. **(C)** Example of a McAdam ellipse with eight vectors in the color triangle. **(D)** Example of a red-green s ine-wave grating.

The CCT is a color discrimination test that uses pseudoisochromatic stimuli in a luminance and spatial noise background (Figure 2A), with stimulus parameters that are optimal for color vision assessment (Mollon and Reffin, 1989; Reffin et al., 1991; Regan et al., 1994; Ventura et al., 2003; Costa et al., 2006). In luminance and spatial noise environment, only the chromatic characteristics of the stimulus change from trial to trial, and therefore, no confounding factors like luminance or contour cues can influence the performance. The stimuli consist of a mosaic of circles of different diameters and luminances forming the background with a subset of circles of a different chromaticity forming a target. The target is a modified Landolt “C” with 1.25° gap for a viewing distance of 2.6 m. Only two parameters vary during the test: (i) the “C” gap appears randomly oriented up, down, left, or right in each trial and (ii) the chromaticity of the target varies along pre-specified vectors in the CIE 1976 *u*′*v*′ color space (Figures 2B,C). Participants are instructed to identify the orientation of the gap in the stimulus by pressing a remote control. A four-alternative forced-choice staircase was used, where for each correct response the chromaticity of the stimulus approached the chromaticity of the background/neutral point (*u*′*v*′: 0.1977; 0.4689) and for each incorrect response it moved away. For each CIE color space vector tested a threshold is calculated by averaging the values of six response reversals (by response reversals we mean one incorrect after one correct response or one correct after one incorrect response). The values averaged are the chromaticity values at the time of the response reversal). The task was terminated after a threshold was calculated for each of the color space vectors tested.

In the CCT, we used two complementary testing protocols that differed in overall duration and chromatic characteristics of the stimuli presented. The Trivector protocol estimates discrimination thresholds for the protan, deutan, and tritan color confusion vectors of the CIE 1976 *u*′*v*′ color space (Figure 2B). The three vectors are tested in random alternation in the same testing session. These confusion lines represent chromaticity values in the color space where subjects with congenital color vision defects are not able to discriminate (Pokorny et al., 1979). Protan stands for reddish, deutan for greenish, and tritan for bluish areas of the color space, stimuli preferentially processed by the L, M, and S wavelength-sensitive cone systems, respectively.

While the Trivector protocol measures thresholds for three vectors in the color space, the ellipses protocol measures thresholds for eight or more vectors around a fixed chromaticity background in the CIE 1976 *u*′*v*′ color space and represent an indicative of the visual system sensitivity to a broad range of hues. The eight vectors are tested two at a time, in random alternation. The vectors here are not the same as in the Trivector test. The eight vectors are separated by 45° so that we can evaluate color discrimination in directions within 360° of the CIE 1976 *u*′*v*′ color space. After the end of the test an ellipse is fitted onto the threshold points in the color space (Figure 2C). The area of that ellipse is considered an indicative of overall color discrimination. Smaller areas mean better discrimination. Another relevant ellipse parameter is the ratio between major and minor axes. A ratio of 1 indicates homogeneous discrimination around the background chromaticity, while a large ratio indicates poor discrimination along a direction in CIE space.

Finally, a Forced-choice Spatial Chromatic CCS was employed to estimate the Red-Green contrast sensitivity for a vertical sine-wave grating of three cycles per degree (Figure 2D, red: *u*′ = 0.288, *v*′ = 0.480; green: *u*′ = 0.150, *v*′ = 0.480). Before starting the CCS task, all subjects underwent a heterochromatic flicker photometry (20 Hz) adjustment to equate perceptually the luminance of the red and green stimuli, thus insuring that individual differences in L and M cone ratio would not influence the results through luminance cues (Mullen, 1985). In the CCS task, the grating started with a contrast value of 4% and chromaticity values according to each subject’s heterochromatic flicker photometry results. We used a two-interval forced-choice psychophysical procedure. Subject’s task was to discriminate the grating from the background chromaticity responding in a remote control if the grating appeared first or second in each trial. A 3 × 1 staircase was used, meaning that the contrast value would decrease 20% after every three consecutive correct responses and increase by 25% for each incorrect response. The test is terminated after six response reversals are obtained and a threshold is calculated by averaging the chromaticity values at the time of the response reversals.

The methods used in this color vision assessment are particularly adequate for a repeated-measures study. Systematic research has shown that learning effects do not affect CCT results after repeated examinations (Costa et al., 2006).

### Data analyses

Analyses of the CCT Trivector Results employed three repeated-measures ANOVA with two within subjects factors: tDCS Stimulation (anodal, cathodal, sham) and Time (During tDCS, 15 min after tDCS). Analyses of the other tests results were performed by separate repeated-measures ANOVA with one within subjects factor (tDCS stimulation). When appropriate, the *post hoc* comparisons were carried using the Fisher LSD test. We measured the effect size using Partial Eta Squared (${\text{η}}_{p}^{2}$) for every ANOVA.

## Results

**Figure 3:**
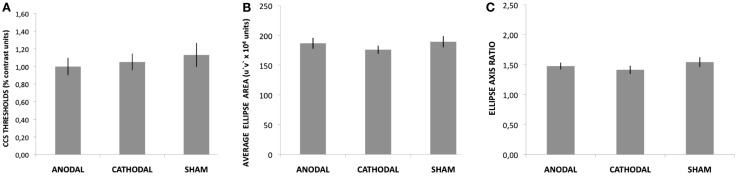
**Results for the CCS task and CCT ellipses test**. None of these comparisons reached the statistical significance criteria established (95%). The bars represent the means and the vertical lines represent SE. **(A)** Average red-green thresholds measured with the CCS task. **(B)** Average ellipse area measured with the CCT ellipses test. **(C)** Average ellipse axis ratio measured with the CCT ellipses test.

The participants reported no adverse effects during or after the stimulation sessions. The ANOVA showed no effects of tDCS on the CCS thresholds [*F*(2, 28) = 1.04, *p* = 0.36, η ${\text{η}}_{p}^{2}$ = 0.08]. This result suggests that only 8% of the variation in threshold values can be attributed to tDCS. The average CCS thresholds were 1.01 (±0.35), 1.05 (±0.33), and 1.13 (±0.32) percent contrast for anodal, cathodal, and sham tDCS, respectively (Figure 3A).

The Analyses of Variance showed no significant effect of tDCS on the average area of the CCT ellipses [*F*(2, 28) = 1.15, *p* = 0.32, η ${\text{η}}_{p}^{2}$ = 0.07] or the ellipses axis ratio [*F*(2, 28) = 1.43, *p* = 0.25, η ${\text{η}}_{p}^{2}$ = 0.09]. The area of the ellipse was on average 186.68 (±35.72), 175.92 (±26.88), and 189.27 (±36.50) *u*′*v*′*10^4^ vector length units for anodal, cathodal, and sham tDCS, respectively (Figure 3B). Average ellipse axis ratios were 1.48 (±0.23), 1.41 (±0.27), and 1.54 (±0.32) for anodal, cathodal, and sham tDCS, respectively (Figure 3C).

**Figure 4:**
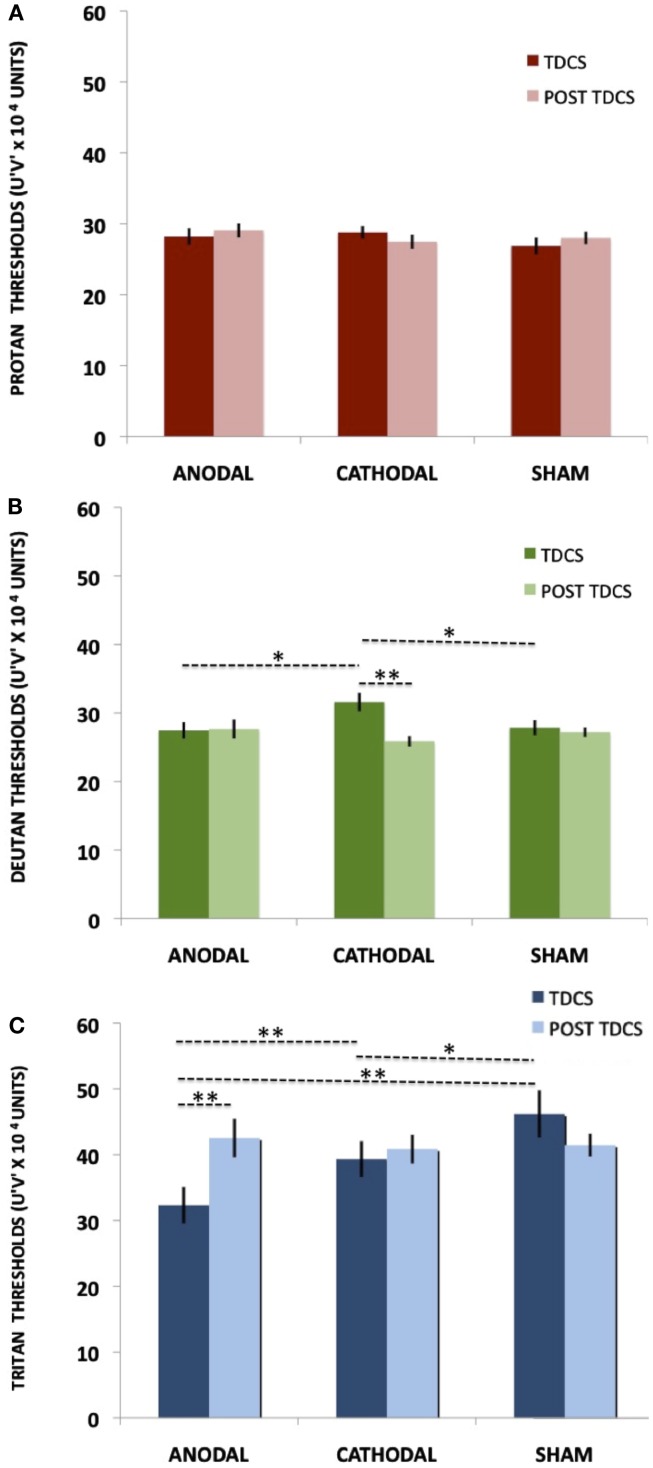
**Online tDCS and Post tDCS comparisons**. The bars represent the means and the vertical lines represent SE. Statistically significant comparisons are marked with asterisks (*p < 0.05 and **p < 0.01). **(A)** Protan threshold values for both tDCS and post tDCS conditions. **(B)** Deutan threshold values for both tDCS and post tDCS conditions. **(C)** Tritan threshold values for both tDCS and post tDCS conditions.

For the protan thresholds, the ANOVA showed no significant effect of tDCS [*F*(2, 28) = 0.66, *p* = 0.52, η ${\text{η}}_{p}^{2}$ = 0.04] or interaction between tDCS and Time [*F*(2, 28) = 0.73, *p* = 0.48, η ${\text{η}}_{p}^{2}$ = 0.04]. Average thresholds measured in *u*′*v*′*10^4^ vector length units for the protan axis were 28.20 (±4.54), 28.80 (±3.43), 26.87 (±4.64) *u*′*v*′*10^4^ for anodal, cathodal, and sham tDCS, respectively (Figure 4A).

For the deutan thresholds, the ANOVA showed no significant effect of tDCS [*F*(2, 28) = 1.12, *p* = 0.33, η ${\text{η}}_{p}^{2}$ = 0.07] and a significant interaction between tDCS and Time [*F*(2, 28) = 5.13, *p* = 0.01, η ${\text{η}}_{p}^{2}$ = 0.26]. When comparing the During tDCS results, Fisher LSD showed significant differences in cathodal vs. sham (*p* = 0.02) and cathodal vs. anodal (*p* = 0.03) comparisons, results that suggest cathodal tDCS impairs deutan discrimination (see Figure 4B; Table 1). No significant differences were found when comparing anodal vs. sham deutan thresholds (*p* = 0.44, Figure 4B). Cathodal vs. post cathodal deutan scores where significantly different (*p* < 0.001), suggesting a return to baseline after 15 min of the end of stimulation (Figure 4B). For the deutan thresholds the averages were 27.47 (±4.69), 31.60(±5.28), and 27.87 (±4.31) *u*′*v*′*10^4^ for anodal, cathodal, and sham tDCS, respectively.

**Table 1:** **Significance values for comparisons of deutan and tritan thresholds**.

	Anodal	Cathodal	Sham	Post anodal	Post cathodal	Post sham
**DEUTAN**						
Anodal	–	0.003	0.444	0.523	0.609	0.732
Cathodal	0.003	–	0.022	0.016	<0.001	0.008
Sham	0.444	0.022	–	0.898	0.206	0.669
Post anodal	0.523	0.016	0.898	–	0.254	0.765
Post cathodal	0.609	<0.001	0.206	0.254	–	0.396
Post sham	0.732	0.008	0.669	0.765	0.396	–
**TRITAN**						
Anodal	–	0.004	«0.001	<0.001	0.001	<0.001
Cathodal	0.004	–	0.045	0.338	0.644	0.521
Sham	«0.001	0.045	–	0.273	0.115	0.260
Post anodal	<0.001	0.338	0.273	–	0.615	0.747
Post cathodal	0.001	0.644	0.115	0.615	–	0.856
Post sham	<0.001	0.521	0.260	0.747	0.856	–

For the tritan thresholds, the ANOVA showed a significant effect of tDCS [*F*(2, 28) = 5.76, *p* < 0.01, η ${\text{η}}_{p}^{2}$ = 0.29] and interaction between tDCS and Time [*F*(2,28) = 7.93, *p* < 0.01, η ${\text{η}}_{p}^{2}$ = 0.36]. *Post hoc* comparisons (Figure 4C; Table 1) showed significant differences in anodal vs. sham (*p* < 0.001), anodal vs. cathodal (*p* < 0.01), cathodal vs. sham (*p* = 0.04), and anodal vs. post anodal (*p* < 0.001). Cathodal vs. post cathodal (*p* = 0.64), post cathodal vs. sham (*p* = 0.11), and post cathodal vs. post sham (0.85) were not significantly different. Thresholds in the tritan axis were on average 32.33 (±10.75), 39.33 (±10.61), and 46.20 (±13.92) *u*′*v*′*10^4^ for anodal, cathodal, and sham tDCS, respectively. The results suggest a reversible improvement in tritan discrimination by tDCS as the thresholds tended to return to baseline after 15 min of the end of stimulation (Figure 4C).

## Discussion

In order to properly discuss our results, a brief review of the organization of visual processing in separate retino-cortical pathways is needed. Human color vision is trichromatic and arises from a comparison of the activation of short (S), middle (M), and long (L) wavelength-sensitive cones: cells with peak sensitivities tuned to light in the “bluish,” “greenish,” and “reddish” portions of the visible spectrum, respectively. Signals from the retinal ganglion cells that compare L and M cone signals project to the Parvocellular (P) retino-cortical visual pathway, while ganglion cells that compare S with combinations of L and M cone signals project to the Koniocellular (K) retino-cortical visual pathway. The P and K pathways are functionally, anatomically, and phylogenetically distinct. Knowledge of primates’ P and K pathways projections from the thalamus lateral geniculate nucleus (LGN) to V1 is robust: P pathway color signals target mostly the 4Cβ (with projections going to layers 4A and 6) while K signals target upper layers 1, 2, 3, and 4A. Although the laminar organization of V1 is well described, state-of-the-art methods have failed to provide a controversy-free picture of the organization of color-coding cells in V1 and some hypothesize that V1 combines part of LGN P and K inputs in arbitrary ways (Conway et al., 2010). Some authors even suggest that interlayer feedbacks and other connectivity peculiarities of V1 completely blur the P and K pathway distinction (see Sincich and Horton, 2005). For reviews on the organization of retino-cortical visual pathways see Callaway (1998, 2005), Hendry and Reid (2000), Xu et al. (2001), Gegenfurtner and Kiper (2003), Briggs and Ursey (2009), and Conway et al. (2010).

The main findings of this study were: (i) anodal tDCS was effective in improving discrimination to the blue (tritan) but did not affect the red-green (protan–deutan) discrimination measured by the CCT and red-green chromatic sensitivity measured by the CCS; (ii) cathodal tDCS had opposite effect on the tritan and deutan thresholds, increasing the sensitivity of the former and decreasing the sensitivity of the latter; (iii) both cathodal and anodal tDCS improved blue discrimination. The main discussion topics will be: (i) possible existence of a ceiling effect limiting the effectiveness of anodal tDCS on the red-green discrimination; (ii) results suggest a functional segregation of P and K pathways in V1.

Converging evidence suggested that this tDCS protocol would be effective to modulate color discrimination. First, as reviewed by Shapley and Hawken (2011), research in the last 25 years shows that V1 plays a critical role in color processing and that it is a much more relevant color-coding center than hypothesized in classic works that discussed modular organization of visual processing. Also, combining the existence of V1 cells that code color and are modulated by luminance signals (Horwitz et al., 2005), the superimposition of color and form processing in the cortex (Johnson et al., 2001; Sincich and Horton, 2005), and the existence of significant effects of tDCS on visual function when using the Oz–Cz montage (Antal et al., 2004a,b,c; Lang et al., 2007; Kraft et al., 2010) suggest that our results are not unexpected.

By using the Oz–Cz electrode montage we intended to particularly modulate the primary visual cortex’s excitability, since it is a superficial cortical area expected to be under Oz electrodes. Placing the return electrode over Cz is particularly adequate for studies of the visual function, since Cz is traditionally used as reference electrode in Visual Evoked Potential studies (i.e., Norcia et al., 1989; Gawne et al., 2011) and pulses of Transcranial Magnetic Stimulation (TMS) over Cz produced no significant BOLD activity in visual areas from V1 to V4 in a concurrent TMS/fMRI study (Ruff et al., 2006). Also, there is substantial evidence that psychophysical response for simple stimuli at threshold levels may closely map the response characteristics of the different visual pathways originated in the retina (Lee, 2011). The abovementioned facts reinforce the adequacy of the methods and rationale employed here to investigate Parvo and Koniocellular pathways cortical organization.

The effect of anodal tDCS on color discrimination in the tritan axis was substantial. Sixty percent of the participants (9/15) had thresholds below 30 × 10^4^ chromaticity difference units (in *u*′*v*′ color space) when receiving anodal tDCS. During the Sham tDCS condition only one participant (1/15 or 6.6%) had tritan threshold values below 30 × 10^4^ units. Costa et al. (2006) tested 36 healthy controls using the same CCT parameters and procedures, but with no tDCS. All participants had tritan discrimination thresholds higher than 30 × 10^4^ units when performing the test binocularly. This shows that anodal tDCS decreased the tritan thresholds to levels that are below normative values. It is noteworthy that anodal tDCS was ineffective on red-green CCS or protan and deutan thresholds, that can also be considered indicatives of the red-green visual discrimination. One possible explanation for that is that koniocellular inputs from the LGN to V1 are more superficial than the parvocellular inputs. We will call this the Layer Hypothesis. On the other hand, the presence of a significant cathodal effect on deutan thresholds speaks against the layer hypothesis since there is apparently no reason why cathodal tDCS would reach layers that the anodal tDCS would not.

The fact that color discrimination is optimal in our subject’s age range can be a determinant of the ineffectiveness of anodal tDCS on red-green discrimination. Previous experiments using tDCS during psychophysical and electrophysiological achromatic contrast sensitivity tests in healthy young adults suggested that ceiling effects could limit the excitatory outcome of the stimulation (Antal et al., 2001, 2004a; Antal and Paulus, 2008). It is also noteworthy that the S cone dominated K pathway is generally more fragile than the P pathway and that acquired color vision defects frequently affect the blue-yellow discrimination more intensely, fact that can be attributed to both structural and functional differences (Pokorny et al., 1979; Gobba and Cavalleri, 2003). Also, thresholds in the protan and deutan axes are generally significantly lower than in the tritan axis (Costa et al., 2006, 2007; Feitosa-Santana et al., 2010). This could also be a determinant of the existence of an anodal tDCS effect on tritan thresholds alone.

The elucidation of the mechanisms behind this proposed ceiling effect remains beyond the scope of the present work. In spite of that, we can say that the existence of a ceiling effect limiting anodal tDCS effectiveness on red-green discrimination is possible. The layer hypothesis cannot be satisfactorily invoked to explain these effects and, as we will discuss in the following paragraphs, it is unclear if there are P and K systems biophysical and morphological differences that could be determinants of this effect. Combining the abovementioned hypotheses with the fact that there is substantial data in the literature showing that a ceiling effect can limit tDCS effectiveness on the visual system (Antal et al., 2001, 2003b, 2004a; Antal and Paulus, 2008) suggest that this can be a real phenomenon relevant to our results and that further research is needed to elucidate the mechanisms behind such effect.

The existence of a qualitatively distinct effect of cathodal tDCS on tritan and deutan thresholds raises more sophisticated hypotheses. Cathodal tDCS is generally expected to impair the performance mediated by the stimulated area (as it did for the deutan discrimination), but in some circumstances, especially when involving discrimination of targets in noisy environments, cathodal tDCS can enhance performance (Antal et al., 2004c). Antal and Paulus (2008) hypothesize that cathodal tDCS can have a distinct effect on the detection of noise and target. Cathodal tDCS would diminish the overall activation level, having a stronger effect on the diffusely responding noise processing cells and therefore increasing signal-to-noise ratio and improving the performance. Although this is a plausible explanation of a performance improvement by cathodal tDCS, it does not account for the opposite effects on tritan and deutan discriminations. This issue is not straightforward and a series of anatomical and biophysical aspects that are yet to be explored can be determinants of this phenomenon. The present work adds relevant information to this debate by showing a rare example of an increase in performance by both anodal and cathodal tDCS in the same task.

The existence of a qualitative difference of cathodal tDCS effects on tritan and deutan discrimination speaks against the Layer Hypothesis. tDCS is optimal for stimulation of superficial brain areas because the maximal current strength is achieved under the electrodes and decreases rapidly at a distance from it (Miranda et al., 2006; Wagner et al., 2007). If cell groups differ only in layer depth, tDCS effects would be only quantitatively different. If the cathodal stimulation reaches the deutan processing cells, anodal stimulation probably reaches these cells too. Therefore, the Layer Hypothesis could help to explain the absence of anodal tDCS effects on red-green discrimination, but not the opposite effects of cathodal tDCS. In order to properly discuss this series of contrasting effects we will have to consider functional, biophysical, and connectivity differences of P and K color-coding cells in V1.

While LGN P and K cells act in a fairly linear way when combining cone inputs, many color-coding V1 neurons act in non-linear ways, and some cone-opponent V1 cells are even influenced by luminance inputs (Hanazawa et al., 2000; Wachtler et al., 2003; Horwitz et al., 2005). De Valois et al. (2000) suggested that approximately half of V1 cells present significant non-linearity in their chromatic responsivity. P and K pathways are not only functionally and anatomically different but they differ in phylogenesis too, with the K pathway being significantly more ancient (Lee, 2011). Considering the functional, anatomical, and phylogenetic differences of P and K pathways, it is possible that morphological and biophysical differences exist and that this could affect tDCS effects. In fact there are morphological and biophysical differences between P and K pathway cells in photoreceptor, bipolar, and ganglion cell layers of the retina, not to mention the LGN. There are also morphological differences between part of the cells that receive P and M (Magnocellular) inputs in V1 (Sincich and Horton, 2005) and in principle different cell types could be distinctively affected by tDCS.

Apart from these, the existence of biophysical and the extent of morphological differences between primate V1 cells receiving P and K inputs is still unclear (Hendry and Reid, 2000; Shostak et al., 2002; Casagrande et al., 2007) and it is still to be discovered if differences at these levels could help to explain the differential effect of cathodal tDCS on deutan and tritan discriminations. Actually, according to Shostak et al. (2002), the morphologic differences between P and K projections from the LGN to V1 seem to be limited to axonal terminal sizes and most of the differences seem to be connectional. These morphologic differences could not fully explain the differential effects of cathodal tDCS. It is likely that P and K inputs in V1 differ mostly in connectivity, since there are several relevant steps of sensory codification between the photoreceptors and V1 and differences at the biophysical level are more likely to be found at the level of the retina or LGN (Shostak et al., 2002; Sincich and Horton, 2005).

It is clear that tDCS is not focal or specific enough to allow definitive conclusions about the nature of the behavioral modulation reported here. Morphological, connectional, or biophysical differences between P and K cells cannot be satisfactorily invoked to account for our results. Notwithstanding, our results are indicative of a functional segregation of P and K cells in V1 and adds relevant information to the debate of whether P and K pathways distinction is blurred at the level of V1. If V1 color-coding cells are organized in myriad ways and the distinction between P and K pathways is blurred after the first synapse in V1 (Sincich and Horton, 2005; Conway et al., 2010), tDCS should affect protan, deutan, and tritan discriminations in a similar way. Our results point to a different direction, suggesting that these pathways can be differentially affected by tDCS. The absence of anodal effects on red-green discrimination can be accounted for by a putative ceiling effect (that reflects functional differences between P and K pathways in V1). The qualitatively different cathodal effects on tritan and deutan discriminations could be accounted for by morphological, biophysical, or connectivity distinctions. Our results suggest a significant segregation between P and K pathways no matter if the determinants are in the molecular or systemic level. The present work shows that tDCS can affect sensory processing in a pathway-specific manner and is an adequate tool to explore the cortical organization of sensory functions.

The anodal tDCS effects on tritan and the cathodal tDCS effects on deutan thresholds tended to return to baseline after 15 min of the end of stimulation. This result is in line with the notion that tDCS has a more limited time course on sensory performance when compared to motor performance (Antal and Paulus, 2008). In addition, tDCS was only delivered once for each current direction in each participant. Current research suggests that in order to achieve stable and long-lasting tDCS effects, more than one session is needed (Zaghi et al., 2010; Brunoni et al., 2011). Future work using tDCS to modulate color perception should approach the issue of the necessary parameters to achieve long-lasting effects of tDCS on this modality. However, the inducement of long-lasting effects on color vision of healthy volunteer is controversial and with ethical implications. At the same time, our findings open an avenue of new investigations. Further studies should focus on the effects of tDCS on color vision defective patients both in terms of acute effect as well as long-lasting effects.

## Conclusion

Our results showed that tDCS can modulate color perception in a pathway-specific robust manner, improving visual discrimination performance to levels that are above the normative values of healthy controls. This suggests that tDCS could have positive outcomes if used for color vision rehabilitation. The distinct effects of tDCS on protan, deutan, and tritan discriminations illustrate that tDCS is an effective tool for the investigation of the cortical organization of visual processing. tDCS had a qualitatively different effect on tritan and deutan discriminations, a result that suggests some level of segregation of P and K pathways within V1. This result adds relevant knowledge to the controversial matter of P and K integration in V1. Future research should target other visual areas involved in color perception. Also, future research combining visual discrimination tasks and tDCS of visual areas should take into account the color parameters of stimuli as possible confounding factors.

## Conflict of Interest Statement

The authors declare that the research was conducted in the absence of any commercial or financial relationships that could be construed as a potential conflict of interest.
